# Super-resolution microscopy reveals γ-secretase at both sides of the neuronal synapse

**DOI:** 10.1186/s40478-016-0296-5

**Published:** 2016-03-31

**Authors:** Sophia Schedin-Weiss, Ina Caesar, Bengt Winblad, Hans Blom, Lars O. Tjernberg

**Affiliations:** Department NVS, Center for Alzheimer Research, Division of Neurogeriatrics, Karolinska Institutet, 141 57 Huddinge, Sweden; Science for Life Laboratory, Royal Institute of Technology, 171 65 Solna, Sweden

**Keywords:** Alzheimer disease, γ-Secretase, Synapse, Hippocampal neuron, Super-resolution microscopy, Stimulated emission depletion (STED) microscopy, Stochastic optical resolution microscopy (STORM)

## Abstract

The transmembrane protein assembly γ-secretase is a key protease in regulated intramembrane processing (RIP) of around 100 type-1 transmembrane proteins. Importantly, it has a pathological role in Alzheimer disease (AD) as it generates the neurotoxic amyloid β-peptide from the amyloid precursor protein (APP). Studies on γ-secretase location are therefore crucial both from a biological and a therapeutic perspective. Despite several years of efforts in many laboratories, it is not clear where in the neuron γ-secretase exerts it’s activities. Technical challenges include the fact that the active enzyme contains four protein components and that most subcellular compartments cannot be spatially resolved by traditional light microscopy. Here, we have used a powerful combination of the two nanoscopy techniques STORM and STED microscopy to visualize the location of γ-secretase in neurons using an active-site specific probe, with a focus on the synapse. We show that γ-secretase is present in both the pre-and postsynaptic compartments. We further show that the enzyme is enriched very close to the synaptic cleft in the postsynaptic membrane, as well as to NMDA receptors, demonstrating that γ-secretase is present in the postsynaptic plasma membrane. Importantly, the expression of γ-secretase increased in the pre- and postsynaptic compartments with the size of the synapse, suggesting a correlation between γ-secretase activity and synapse maturation. Thus, our data shows the synaptic location with high precision in three dimensions and settles the long-lasting debate on the synaptic location of γ-secretase.

**Electronic supplementary material:**

The online version of this article (doi:10.1186/s40478-016-0296-5) contains supplementary material, which is available to authorized users.

## Introduction

γ-Secretase is a key player in the pathology of Alzheimer disease (AD) as it catalyzes the final step in the processing of the amyloid precursor protein (APP) leading to the formation of the neurotoxic amyloid β-peptide (Aβ), which is the major constituent of the amyloid plaques in the brains of AD patients [1]. γ-Secretase generates Aβ of variable lengths, and especially the 42 and 43 amino acids variants (Aβ42 and Aβ43) are prone to form neurotoxic oligomers. Intracellular levels of Aβ42 are increased in hippocampal neurons in AD, accumulate in synapses and correlate with AD pathology [2, 3]. Direct inhibition of γ-secretase as a treatment strategy for AD is complicated by the fact that this enzyme has around 100 different substrates in addition to APP, including Notch. Importantly, Aβ has been shown to be produced at the synapse, and whether this occurs pre- or postsynaptically is debated [4–6]. This uncertainty is partially due to the technical challenge involved in determining the synaptic substructures, which are considerably smaller than the resolution obtained by traditional fluorescence microscopy. In addition, since γ-secretase consists of four protein components, presenilin, nicastrin, anterior pharynx-defective phenotype 1 (Aph-1) and PS-enhancer 2 (Pen-2) [7], it is desirable to study the active enzyme and not, as in most previous studies, the individual components. Thus, detailed investigations of the subcellular locations and regulatory mechanisms of γ-secretase activity are instrumental for elucidation of AD pathogenesis.

Advances in fluorescence microscopy have pushed the resolution limits to allow more than ten times improved resolution compared to confocal microscopy. Two recent techniques are stochastic optical reconstruction microscopy (STORM) [8] and stimulated emission depletion (STED) microscopy [9]. Both have their inherited strengths and limitations. STORM takes advantage of the fact that certain fluorophores can cycle between dark and fluorescent states. When the sample is illuminated with an activation laser and/or a reporter laser, a series of images are acquired where only a sparse set of fluorophores are switched ON to give a signal in each camera frame. Combining data from all individual camera frames allows calculation of the position of each fluorophore with nm precision. STED microscopy uses a similar switching approach to break the diffraction barrier [9]. By superimposing a donut-shaped depletion beam on the excitation beam, fluorophores around the excitation spot in a specimen can be switched OFF by stimulated emission depletion. Thus, while scanning over a sample area, the emitting spot in each targeted position is narrowed to allow resolution down to ~20 nm in the lateral plane.

To study the synaptic localization of γ-secretase in hippocampal neurons in detail, we used a probe that binds selectively to active γ-secretase and visualized the cellular location with a unique combination of microscopic techniques. Initial experiments showed enrichment of γ-secretase at synapses. The precise synaptic location was therefore studied with STORM to visualize the pre- and postsynaptic location in three dimensions and multi-colored STED, revealing that γ-secretase is located in both pre- and postsynaptic compartments of the neuronal synapse, and that the distribution of γ-secretase differs in different stages of synapse maturation.

## Materials and methods

### Cell culturing

Mouse hippocampal neurons were cultured in 35 mm glass bottom dishes (No 1.5 P35G-1.5 10-C, MatTek Corp, Ashland, USA) essentially as described earlier [10, 11]. Briefly, cells were isolated from the dissected hippocampi and cortices of embryonic (E16.5) C57BL6 mice. The mice used in this study for preparation of primary neuronal cultures were treated according to the Karolinska Institutet’s as well as the national guidelines and the study was approved by the animal research ethical committee in southern Stockholm (ethical permit S154-12). The animals were sacrificed by servical dislocation and no experiments were performed on live animals.

The hippocampal neurons were grown on the cover glass in the center of the plates with a support layer of cortex neurons in neurobasal medium of which half the volume was replaced by fresh medium once per week. After 21 days in vitro (DIV) the cells were fixed in neutral-buffered 4 % (*w*/*v*) paraformaldehyde solution (Sigma-Aldrich) for 10 min at room temperature (RT), stored in DPBS at 4 °C and labeled for immunocytochemistry studies. Immediately prior to staining, the cells were permeabilized in 0.4 % CHAPSO. 

### Conjugation of antibodies

Conjugation of antibodies to a fluorophore (for confocal microscopy, STED and d STORM) or two fluorophores (for STORM) was performed essentially according to the STORM sample preparation protocol supplied by Nikon, using the succinimidyl esters of the fluorophores. Streptavidin (A1495,0005, AppliChem) was conjugated to Alexa594 (A20004, Life Technologies) for confocal microscopy and STED, or with Alexa647 for dSTORM. For STORM, streptavidin was conjugated to Cy3 (activator) and Alexa647 (reporter). Donkey anti-mouse IgG (A16019, Life Technologies) for STORM was labeled with Alexa405 as activator and Alexa647 as reporter.

### Studies on γ-secretase localization in axons and dendrites

γ-Secretase was studied by immunohistochemistry and confocal microscopy, by using Tau as axonal marker and MAP2 as dendritic marker. γ-Secretase was labeled with an active site γ-secretase inhibitor (L-685.458) with a transferable biotin group and a photoreactive group (GTB) as described earlier [11]. Fixed and permeabilized cells were blocked with 10 % normal goat serum (NGS) in PBS for 10 min at RT and, to avoid unspecific binding of streptavidin, with avidin/biotin (Vector Laboratories) or streptavidin/biotin (Life Technologies) according to the manufacturers recommendations. A combined blocking/preincubation step was first performed using 10 μM (Z-LL)_2_ Ketone (421050, Calbiochem) in the biotin solution to block binding sites for signal peptide peptidase (9). The cells were then incubated with 200 nM GTB, 10 μM (Z-LL)_2_ ketone and polyclonal rabbit anti-MAP2 IgG diluted 1:1000 (AB5622, Millipore) in 3 % NGS at 4 °C ON. For control experiments testing the specificity of the GTB binding, 10 μM γ-secretase inhibitor L685,458 or 10 μM JC18 were added in the last blocking/preincubation step and in the first incubation step. Next, cells were washed 2 × with PBS, followed by UV illumination for 10 min on ice to crosslink GTB via photoactivation [11]. After washing for 5 × 2 min, a secondary incubation step was conducted for 1 h at 37 °C in 3 % NGS containing Alexa594-conjugated streptavidin (~30 nM, conjugated in-house) and Alexa488-conjugated mouse monoclonal anti-Tau-1 IgG diluted 1:100 (MAB 3420A4, Millipore). The final washing step was conducted for 3 × 5 min in 0.1 % CHAPSO in DPBS, 3 × 5 min in DPBS and 1 × 5 min in water. The cells were mounted with ProLong gold antifade reagent (P36930, Life Technologies). Images were acquired in sequential mode on a Nikon A1RSi or A1+ point scanning confocal inverted microscope using a 60× oil immersion objective with an image size of 1024 × 1024 pixels. Excitation lasers were 488, 561 and 640 nm and a pinhole size corresponding to 1 airy unit (at 561 nm) was used.

### Preparation of samples for STORM imaging

Fixed primary hippocampal neurons were treated with 0.1 % of the reducing agent NaBH_4_ (prepared immediately before use) for 7 min at RT prior to permeabilization in 0.4 % CHAPSO. Blocking steps were done essentially as described above for confocal microscopy. In the first antibody incubation step, a combination of either of mouse anti-synaptophysin IgG diluted 1:1000 (SP15, Enzo Life Sciences), mouse anti-PSD95 IgG diluted 1:100 (Ab2723, Abcam) or mouse anti-NMDAR2B (610416, BD Transduction Laboratories) with 10 μM (Z-LL)_2_ ketone and 200 nM GTB ± 10 μM L685,458 were used. The second incubation buffer, used after washing and UV illumination, contained Cy3/Alexa647-conjugated streptavidin (~30 nM) and Alexa405/Alexa647-conjugated anti-mouse IgG diluted 1:100 in 3–5 % NGS. Washing (conducted as in sample preparation for confocal microscopy) was followed by postfixation in 3 % formalin/0.1 % glutaraldehyde for 10 min at RT, followed by washing 3 × 5 min with DPBS and 1 × 5 min with water. Negative controls lacking either of the primary antibodies or GTB were also prepared.

### STORM imaging

STORM imaging was performed on a Nikon point scanning confocal A1+Si Inverted microscope equipped with TIRF, STORM and an Andor EM-CCD camera. A 100 × (NA 1.55) objective was used for STORM acquisition. NIS-Elements AR 4.30.02 software was used and emission was measured after excitation with a 647 nm laser. dSTORM was used for imaging of one-color samples and was performed with a one-channel setting with a 647 nm laser for the emission and with the addition of 405 nm laser activation pulses (to enhance the blinking rate by pushing the reporter fluoropohore to the dark state). Activator-based STORM data acquisition was performed recording the emission obtained by the 647 nm laser, according to the Nikon N-STORM operation manual (M605E 13.12 Nx.4). Two-channel settings with 405 and 561 nm activation lasers were used. By the additional presence of an activator fluorophore on each fluorescently labeled probe to push the reporter back to the dark state, the speed of the blinking is increased. Since the reporter fluorophores may blink even in the absence of activator, the activator laser light to some extent affects the blinking speed even in the absence of activator fluorophore. Negative controls lacking either the γ-secretase probe GTB or the synaptic marker antibody to measure potential cross-talk between the channels were thus also analyzed. Imaging cycles were set to contain one activation frame (i.e. image acquired directly after an activation laser pulse) followed by four reporter frames (i.e. images taken of the reporter emission), to be able to differ between probe-specific and unspecific signals [12]. Only the detected signals in the first frame were used for localization calculations of the super-resolved STORM images. During the long data acquisition, the number of detected signal events successively decrease in the cycles, containing the four consecutive reporter frames following the activation frame, due to photobleaching. The activation laser power was therefore increased during the acquisition via the auto Laser Power function to keep the number of blinks in the consecutive cycles at similar level throughout the whole acquisition. Acquisition was conducted for up to 7000 frames and the frame speed was set to the highest possible value (1 frame), i.e. 9–10 ms/frame. Calibration curves for 3D STORM analyses was done using 0.1 μm TetraSpeck Microspheres (T-7279) as recommended by Nikon in the STORM manual.

The xy resolution in a STORM image is related to the diameter of each Gaussian fit: more photons detected at a spot generates a smaller Gaussian fit to the point spread function, giving a higher probability of accurately determining the Gaussian center. This value is variable from spot to spot, and therefore the resolution differs both within and between samples. Precautions to minimize this spot were taken for instance by calibrating the instrument and performing postfixation after staining. In our STORM images, the molecule localization precision were between 2 and 6 nm for all spots and we were able to separate spots with a distance of down to 6 nm. In the Z directions, based on the objective and the 3D astigmatic lens, we are able to segment down to about 50 nm with good results thanks to the calibration curve, with localization precisions in Z of about 20 nm.

Data analysis was done by using the NIS-Elements AR analysis 4.40.00 software using settings recommended in the Nikon N-STORM operation manual (M605E 13.12 Nx.4). The frames from around number 1000 up to 6000–7000 were included in the analysis. Gaussian rendering of localization coordinates was performed with a minimum Gaussian rendering threshold set to 2 nm. Quantification of the number and area of the dots in each channel of the STORM images were performed from full resolution screenshots of the STORM images saved as tiff images with the full bit depth and using General Analysis in NIS Elements (Nikon). GTB signals were calculated from 60 PSD95 cluster in one STORM image and from 66 synaptophysin clusters in another STORM image. The number of dots from GTB signals were plotted versus the number of dots from synaptophysin or PSD95 signals using GraphPad Prism 5.0d (GraphPad Software, Inc.). The background of the GTB signals, calculated as the mean values ± SD from 10 synaptophysin or PSD95 clusters in samples that were incubated without GTB, were set as threshold values for γ-secretase-containing synapses.

### Preparation of samples for STED and confocal imaging

Pre- and post-synaptic localization of γ-secretase was studied by immunohistochemistry and STED microscopy, using synaptophysin or synaptobrevin as presynaptic marker and PSD95 as postsynaptic marker. γ-Secretase was labeled with GTB and Phalloidin was used as a structural marker of the axons and dendrites. Fixation, permeabilization and blocking were performed as described above for STORM analyses. For presynaptic triple staining; the cells were incubated with 200 nM GTB and mouse monoclonal synaptophysin antibody diluted 1:200 (ADI-VAM-SV011-F, Enzo) in 3 % NGS at 4 °C ON. For postsynaptic triple staining; the cells were incubated with 200 nM GTB and mouse monoclonal PSD95 antibody diluted 1:200 (ab18258, Abcam) in 3 % NGS at 4 °C ON. Co-staining of the γ-secretase components PS1 or nicastrin with GTB in the synapses were studied by confocal microscopy in samples prepared in a similar manner as the triple-stained samples for STED, with GTB and polyclonal rabbit Nicastrin antibody diluted 1:100 (N1660, Sigma) or polyclonal rabbit PS1-NTF antibody diluted 1:100 (529591, Calbiochem). For pre- and postsynaptic quadruple staining; the cells were incubated with 200 nM GTB, rabbit polyclonal synaptobrevin antibody diluted 1:200 (104203, Synaptic System) and mouse monoclonal PSD-95 antibody diluted 1:200 (ab18258, Abcam) in 3 % NGS at 4 °C ON. The cells were rinsed 2 × in DPBS followed by a 5 min incubation in DPBS at RT. The cells were UV illuminated for 10 min on ice to crosslink GTB via photoactivation followed by a 5 × 2 min of washing in 0.1 % Tween-20 in DPBS. For both pre- and postsynaptic triple staining; a second incubation step was performed with ATTO594-conjugated streptavidin diluted 1:200 (S32354, Invitrogen), TRITC-conjugated Phalloidin diluted 1:100 (P1951, Sigma-Aldrich) and AbberiorSTAR635-conjugated anti-mouse antibody diluted 1:200 (2-0012-007-2, Abberior) in 3 % NGS at 37 °C for 1 h. For pre- and postsynaptic quadruple staining; the cells were incubated with Alexa488-conjugated Phalloidin diluted 1:100 (A12379, Life Technology), TRITC-conjugated anti-rabbit antibody diluted 1:200 (T2769, Invitrogen), ATTO594-conjugated streptavidin diluted 1:200 (AD 594-61, Atto-Tech) and AbberiorSTAR635-conjugated anti-mouse antibody diluted 1:200 (2-0012-007-2, Abberior) in 3 % NGS at 37 °C for 1 h. The final washing step was 3 × 10 min in 0.1 % Tween-20 in DPBS, 1 × 5 min in DPBS and 1 × 5 min in water. The cells were mounted with ProLong gold antifade reagent (P10144, Life Technologies).

### STED imaging

Part of the Stimulated Emission Depletion (STED) imaging was performed with a commercial Leica SP5 STED microscope [13]. The dual-color setup feature two pulsed diode lasers (PDL 800-B, PiqoQuant) for excitation at 532 and 640 nm and tunable near-infrared pulsed depletion laser (MaiTai, Spectra Physics). A 100×/1.4 NA chromatically red-shifted oil immersion objective (HCX PL APO STED, Leica Microsystems) was used for super-resolution imaging. Fluorescence signals were passed through a 0.8–0.9 Airy unit pinhole, a dichroic mirror and separate bandpass filters (582/75 and 685/40 from Semrock) placed in front of two avalanche photodiodes. Dual-color frames (1024 × 1024) were acquired sequentially line-by-line at scan speed of 600 lines per second with a pixel size of 25 nm. Each frame consisted of 6 line averages and required ~10 s recording time. The setup was additionally applied to detect a third confocal channel (i.e. in the blue spectra) to visualize neurite morphology.

Additionally a Leica SP8 STED X system with three STED lasers for depletion of fluorophores emitting in the blue/green (592 nm, MPB Communications Inc), orange (660 nm, Laser Quantum, USA) and red/far-red (775 nm, OneFive GmbH) was used. A single depletion wavelength was applied for multi-color STED imaging. For excitation a tunable pulsed white light fiber laser emitting between 470 and 670 nm was used. A 100× chromatically optimized oil immersion objective (HC PL APO 100×/1.40 OIL STED WHITE, Leica Microsystems) was used for confocal and STED imaging. Fluorescence signals were passed through a 0.8–0.9 Airy unit pinhole, dichroic mirrors optimized for each STED laser and notch filters placed in front of sensitive photodetectors (Leica Hybrid Detectors). Multi-color frames (1024 × 1024) were acquired sequentially frame by frame at a scan speed of 600 lines per second with a pixel size of 20 nm. Each frame consisted of 6 line averages and required ~10 s recording time. All STED images are presented as raw data.

Precision calibrations were done by using 40 nm fluorescent beads (FluoSpheres, Invitrogen) spin-coated onto the glass surface of a coverslip and mounted in Mowiol. Resolved lateral FWHM-values of 42 +/− 3 nm (*n* = 30) was achieved on both systems (exc. 635 nm and depletion 750 to 775 nm), pointing to STED focal widths of sub 40 nm. Precision calibrations for 3D-STED generated, with the same bead sample as above, FWHM-values of 119 +/− 4 nm (*n* = 15). DNA-origami spaced 44 nm (STED 44R, Gattaquant Germany) was after bead calibration furthermore resolved on both systems FWHM 45 +/− 2 nm (*n* = 25).

Quadruple stained STED micrographs were calculated for total fluorescence intensity and mean fluorescence intensity per pixel of 70 pre/postsynaptic pairs by LAS AF Version 2.6.3 build 8173 Software (Leica Microsystems CMS GmbH). Background fluorescence was subtracted and fluorescence intensity of GTB versus synaptobrevin or PSD95 was plotted by GraphPad Prism 5.0d (GraphPad Software, Inc.).

## Results

We have studied the presence of the Alzheimer disease-related enzyme γ-secretase in axons, dendrites and synapses of mouse primary hippocampal neurons. For selective labeling of γ-secretase, we used a newly developed probe (GTB), which contains the active site inhibitor L685,458 coupled to a spacer, a photo-reactive group and a biotin group [11] in combination with fluorescently labeled streptavidin. We have previously shown the specificity of this probe for γ-secretase by competition experiments and titrations [11]. To further verify the specificity we have in this study showed that GTB can be competed also by other active site inhibitors, as exemplified here with JC18 (Additional file 1: Figure S1). We further showed that competition of GTB by L-685,458 works also in STORM experiments (Additional file 1: Figure S2). In addition to GTB, we stained neurons with axonal, dendritic and synaptic markers and analyzed the cellular and synaptic location with confocal microscopy and the two super-resolution microscopy techniques STORM and STED.

### Presence of γ-secretase in axons and dendrites

**Fig. 1:**
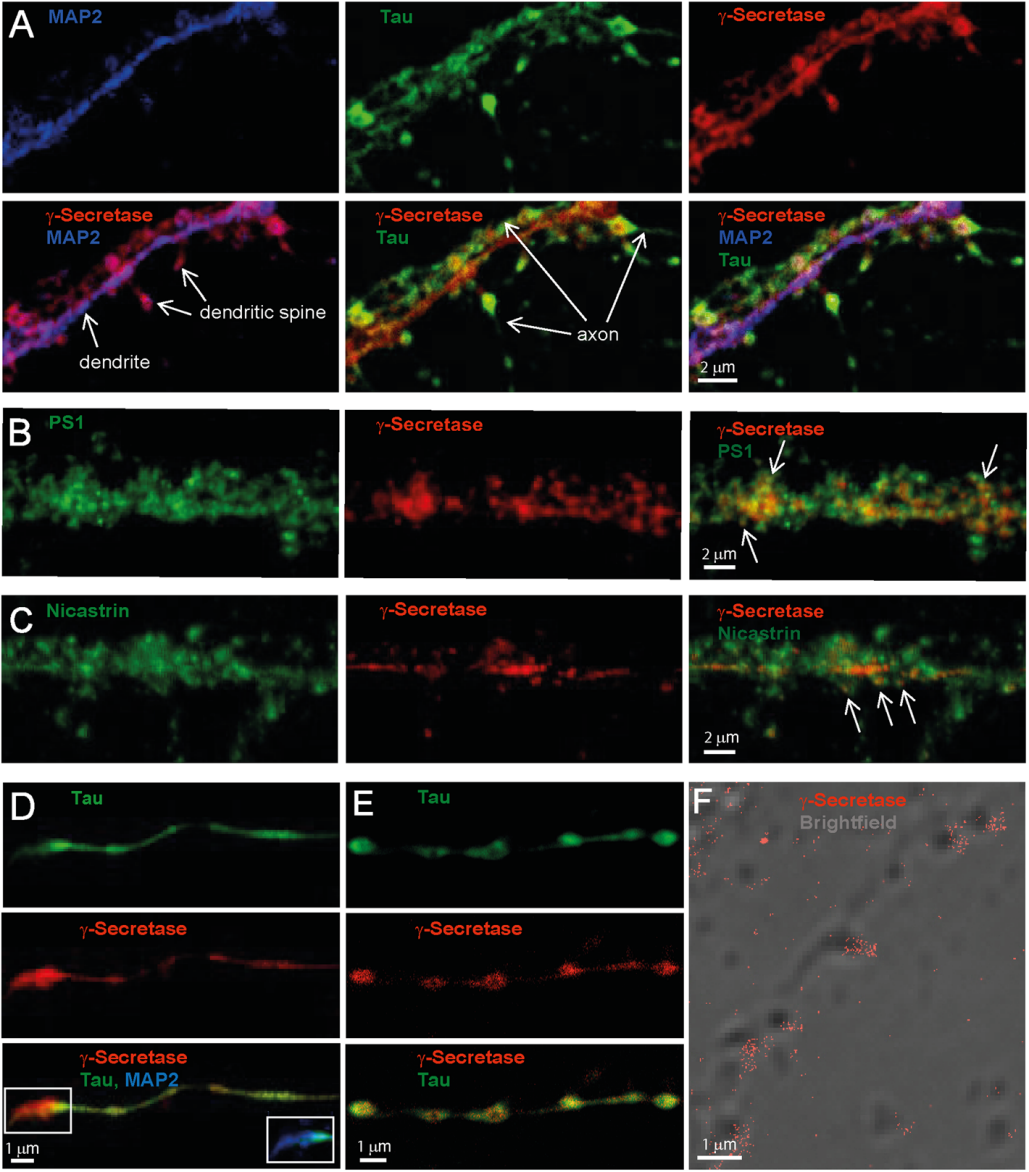
Localization of γ-secretase in axons, dendrites and spines of mouse primary hippocampal neurons analyzed by confocal and STED microscopy as well as dSTORM. γ-Secretase was stained with GTB in combination with streptavidin conjugated with either Alexa594 (confocal and STED microscopy) or Alexa647 (confocal and dSTORM). (**a**) Confocal image of a zoomed dendrite stained for MAP2 (*blue*), surrounding axons stained for tau (*green*) and γ-secretase (*red*). The individual stains are displayed in the *upper panel*, while different combinations of merged images are shown in the *lower panel*, as indicated. (**b**) Confocal image of PS1 and γ-secretase labeling in a close-up of a dendrite. *Arrows* point at synaptic regions with co-staining of PS1 and γ-secretase (*yellow*). (**c**) Confocal image of PS1 and nicastrin labeling in a close-up of a dendrite. *Arrows* point at synaptic regions with co-staining of nicastrin and γ-secretase (*yellow*). (**d**) Confocal image of an axon from the same cell as in (**a**) with tau staining in *green* in the *upper panel*, γ-secretase in *red* in the *middle panel* and a merged image in the *bottom panel*. The *inset* shows a zoomed portion of the merged image with the addition of MAP2 (*blue*). (**e**) A combination of confocal (for tau, *upper panel*) and STED microscopy (for γ-secretase, *middle panel*) was used to visualize the enrichment of active γ-secretase in the axonal boutons. A merged image is shown in the *bottom panel*. (**f**) dSTORM image of active γ-secretase (*red*) overlayed with a brightfield image. Note enrichment of active γ-secretase at spines

Our initial experiments showed that γ-secretase to a large extent was localized to neurites and synapses. Thus we focused our work on these structures and the pre- and postsynaptic compartments. γ-Secretase was visualized by staining the neurons with GTB and Alexa594-streptavidin, and fluorescently labeled antibodies directed to the microtubule-binding proteins Tau and MAP2 were used as axonal and dendritic markers, respectively [10]. Confocal microscopy showed that the dendrites formed several synapses with axons running in parallel with, as well as between, dendrites (Fig. 1a), and γ-secretase was found both in axons and dendrites, and was enriched in the synapses (Fig. 1a). Staining of PS1 and nicastrin showed that these components were found in the same regions as GTB and also in other regions (Fig. 1b and c). This is expected, since only a portion of neuronal PS1 and nicastrin molecules are located to the fully assembled enzyme complex. In axons that grew individually, i.e. to a high extent separated from dendrites, γ-secretase was enriched in pre-boutonal structures (Fig. 1d). The enzyme was also enriched in the axon terminal synapse (Fig. 1d, lower panel, inset). A STED image of γ-secretase, with co-labeling of Tau protein as axonal marker, further verified that γ-secretase was enriched in the pre-boutons and showed that the γ-secretase staining was most intense in the outer parts of these (Fig. 1e). Enrichment of γ-secretase at spines was further confirmed by a brightfield image overlayed with direct STORM (dSTORM), in which γ-secretase was stained by GTB and streptavidin labeled with the reporter fluorophore Alexa647 (Fig. 1f). Thus, three different microscopic techniques showed that γ-secretase is enriched at the neuronal synapse.

### Two-color 3D STORM

The enrichment of γ-secretase at the synapse lead us to investigate whether the enzyme is present in the pre- or postsynaptic compartments, which are separated by a cleft that is only 20–25 nm wide [12]. This distance is around ten-fold smaller than the resolution obtained by traditional confocal microscopy, and two-color STORM was thus used to better visualize synaptic locations. γ-Secretase was labeled with GTB and an activator/reporter set of Cy3/Alexa647-conjugated streptavidin. Antibodies directed to synaptophysin, a synaptic vesicle protein [14], or PSD95, a protein found in the postsynaptic density of dendritic spines [15], were used as pre- and postsynaptic markers, respectively, and were labelled with an Alexa405/Alexa647-conjugated secondary antibody. The specificity of the γ-secretase labeling was determined in a competition experiment by using a 50-fold molar excess of the free γ-secretase inhibitor L685,458, showing that the majority of γ-secretase staining was competed by the free inhibitor (Additional file 1: Figure S2a and b). To minimize potential cross-talk between the channels, a measurement cycle comprising 4 reporter frames (images acquired for the reporter emission) after each activator frame (image taken directly after one activation laser pulse) was used. Only the signals in the first reporter frame were counted as specific signals. With this approach the cross-talk between the channels was very low in negative controls lacking either γ-secretase or the synaptic marker (Additional file 1: Figure S2a, c and d).

**Fig. 2:**
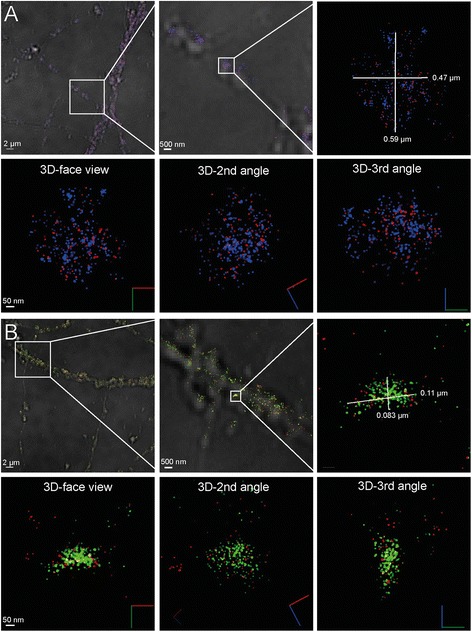
STORM imaging of γ-secretase in synapses. (**a**) Localization of active γ-secretase at the presynaptic zone showing γ-secretase (*red*) and the presynaptic marker synaptophysin (*blue*). The *upper left panel* shows a typical STORM image on a brightfield overlay to the left, with increased zooming in the *middle* and *right panels*, displaying an individual synaptic region to the right. The *lower panel* shows the same synapse displayed in 3D view from three different angles where *red*, *green* and *blue lines* represent X, Y and Z directions, respectively. (**b**) Localization of active γ-secretase at the postsynaptic zone showing γ-secretase (*red*) and the postsynaptic marker PSD95 (*blue*). The *upper left panel* shows a typical STORM image on a brightfield overlay to the left, with increased zooming in the *middle* and *right panels*. The *lower panel* shows the same synapse displayed in 3D view from three different angles where *red*, *green* and *blue lines* in the symbol represent X, Y and Z directions, respectively

In agreement with its presence in synaptic vesicles in presynaptic zones of axons, synaptophysin was distributed in concentrated areas (Fig. 2a). The majority of the presynaptic areas contained γ-secretase, and γ-secretase was found to be distributed throughout the synaptophysin-positive region (Fig. 2a). The mathematically fitted Gaussian distribution of signals from individual fluorophores (measured as the width at half of the maximum intensity), varied between 2 and 6 nm, indicating high quality of the staining as well as precise calibration of the microscope. Thus, single molecule detection with ultra-high precision is enabled. The projected size of the synaptophysin-stained area in a typical synapse measured around 0.5 × 0.6 μm in the x-y direction (Fig. 2a, upper panel). Since we conducted STORM in three dimensions, we could study the distribution of synaptophysin and γ-secretase from different angles (Fig. 2a, lower panel, Additional file 2: Video S1). Proximity between γ-secretase and synaptophysin was apparent from all angles, and the distance observed between the synaptophysin and γ-secretase signals were in some cases less than 10 nm.

The staining of γ-secretase and synaptophysin was evenly distributed in some presynapses, whereas others showed clusters of both synaptophysin and γ-secretase (Additional file 1: Figure S3a-c). Dense labeling of synaptophysin indicates clusters of synaptic vesicles docked to the plasma membrane in the active zone of the presynapse [16]. Interestingly, the lack of γ-secretase staining in these clusters suggests that γ-secretase is absent in docked synaptic vesicles. A high intensity γ-secretase staining was in some synapses observed closely outside the presynaptic area (Additional file 1: Figure S3c and Additional file 3: Video S3). A distance of about 70–80 nm between synaptophysin at the active zone and γ-secretase suggests that γ-secretase is located also at the postsynaptic side.

The postsynaptic marker PSD95 stained more dense and smaller areas than synaptophysin (Fig. 2b), as expected from its presence in the postsynaptic density, and γ-secretase was present in proximity to this staining [12]. A typical PSD95 region (measuring 0.08 × 0.1 μm in the x-y direction) containing γ-secretase is shown in Fig 2b, upper left panel. The presence of γ-secretase close to the PSD was apparent also when the images were viewed from different angles (Fig. 2b, Additional file 4: Video S2). γ-Secretase and PSD95 distribution varied between different synapses (Additional file 1: Figure S3d-f). In some cases, two or more PSD95 regions were found in close proximity, suggesting that they, in agreement with previous observations [15], were located in the same dendritic spine (Additional file 1: Figure S3e and Additional file 5: Video S4). In addition to PSD95-rich regions that contained high levels of γ-secretase, some PSD95-rich regions that lacked γ-secretase were observed (Additional file 1: Figure S3f). Five out of 60 quantified PSD95 clusters had GTB signals below the threshold value determined from the mean of the background staining for GTB, suggesting that less than 8 % of postsynapses lacked γ-secretase (Additional file 1: Figure S6).

### STED analysis of γ-secretase at the synapse

**Fig. 3:**
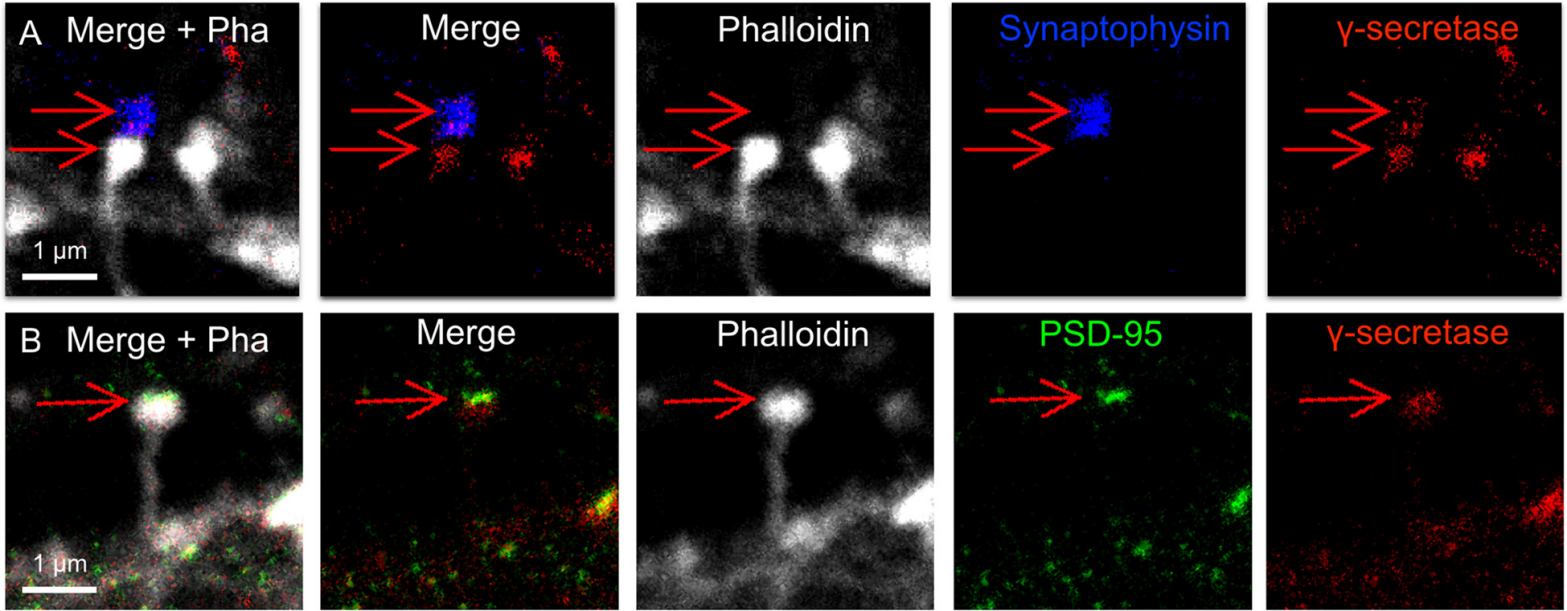
STED imaging of γ-secretase in synapses with triple-staining. γ-Secretase and the synaptic markers were visualized to show the synaptic location of γ-secretase together with f-actin to outline the cellular structure. (**a**) Localization of γ-secretase (*red*) at the synapse using synaptophysin (*blue*) as a presynaptic marker. Dendritic spine structure was visualized with Phalloidin as a marker for f-actin (*white*). The distribution of γ-secretase throughout the synaptophysin-stained region is marked by the *upper arrow*, whereas the γ-secretase staining in the dendritic spine is shown by the *lower arrow*. (**b**) Localization of γ-secretase (*red*) at the synapse using PSD95 (*green*) as a postsynaptic marker and phalloidin as a marker for f-actin (*white*). The presence of γ-secretase in the postsynaptic compartment is shown, where the postsynaptic marker PSD95 is found in dense regions inside the dendritic spine head (*arrow*)

To further investigate the synaptic location of γ-secretase, we labeled γ-secretase (GTB and Atto594-conjugated streptavidin), f-actin for neuronal structure (phalloidin conjugated to tetramethylrhodamine (TRITC), and either synaptophysin or PSD95, and analyzed the samples by multi-color STED. The pre- and postsynaptic markers were detected with an AbberiorSTAR635-conjugated secondary antibody. γ-Secretase was present within the synaptophysin-stained region and the staining was unevenly distributed, in line with clustering of γ-secretase within the presynaptic compartment (Fig. 3a). The co-staining of γ-secretase, PSD95 and f-actin confirmed that γ-secretase was also present in the dendritic spine, with a more dense γ-secretase staining in the postsynaptic than in the presynaptic region (Fig. 3b).

There was a large variation of γ-secretase staining in different presynapses as well as in different postsynapses (Additional file 1: Figure S4a-g). In some cases, the distribution of γ-secretase was very similar to that of PSD95, suggesting that γ-secretase can be enriched at the postsynaptic density. In other cases, γ-secretase was present in the entire dendritic spine head. Most spines showed only one PSD95 region, but some had more than one cluster of PSD95 [15]. In certain cases, two spines containing γ-secretase were found in very close proximity, suggesting that they were connected to the same presynapse and, in addition, two spine heads sharing the same shaft were observed (Additional file 1: Figure S4d-g).

**Fig. 4:**
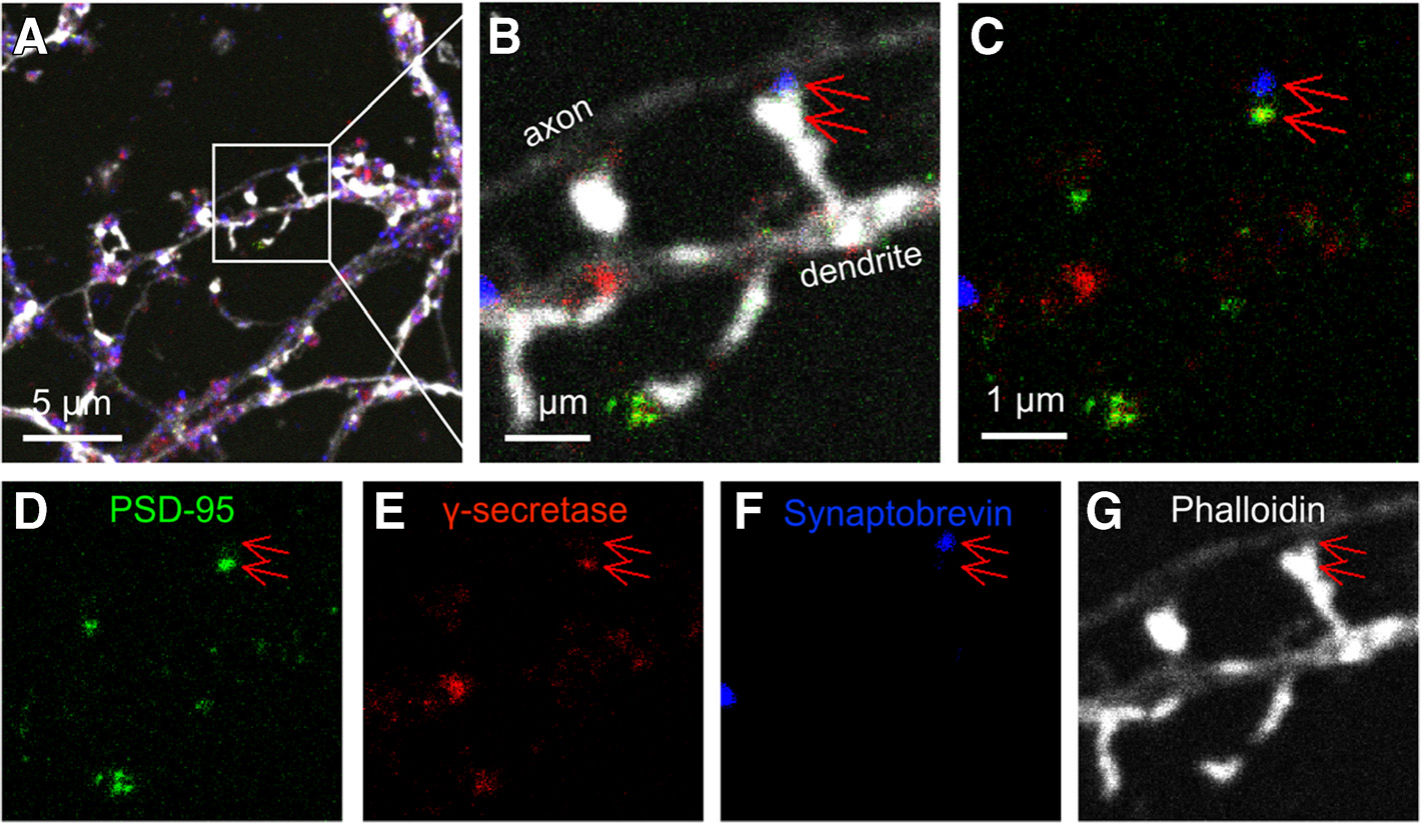
STED imaging of γ-secretase in synapses with quadruple-staining. (**a**) γ-Secretase (*red*), postsynaptic PSD95 (*green*) and presynaptic synaptobrevin (*blue*). Neuronal structure is visualized by f-actin in an overlaid non-STED confocal channel (*white*). (**b**) A zoomed axon and dendrite from (a) indicating an active dendritic spine with the presynapse and postsynapse, indicated by synaptobrevin in *blue* (*upper arrow*) and PSD95 in *green* (*lower arrow*) respectively. (**c**) Merged image of (b) without Phalloidin indicating that γ-secretase (*red*) is mainly found in the postsynapse (*lower arrow*), co-localized with PSD95 (*green*). Small amounts of γ-secretase (*red*) is found in the presynapse (*upper arrow*), co-localized with synaptobrevin (*blue*). Individual channels of (**d**) Postsynaptic marker PSD95, (**e**) γ-Secretase, (**f**) Synaptobrevin and (**e**) Confocal image of f-actin

**Fig. 5:**
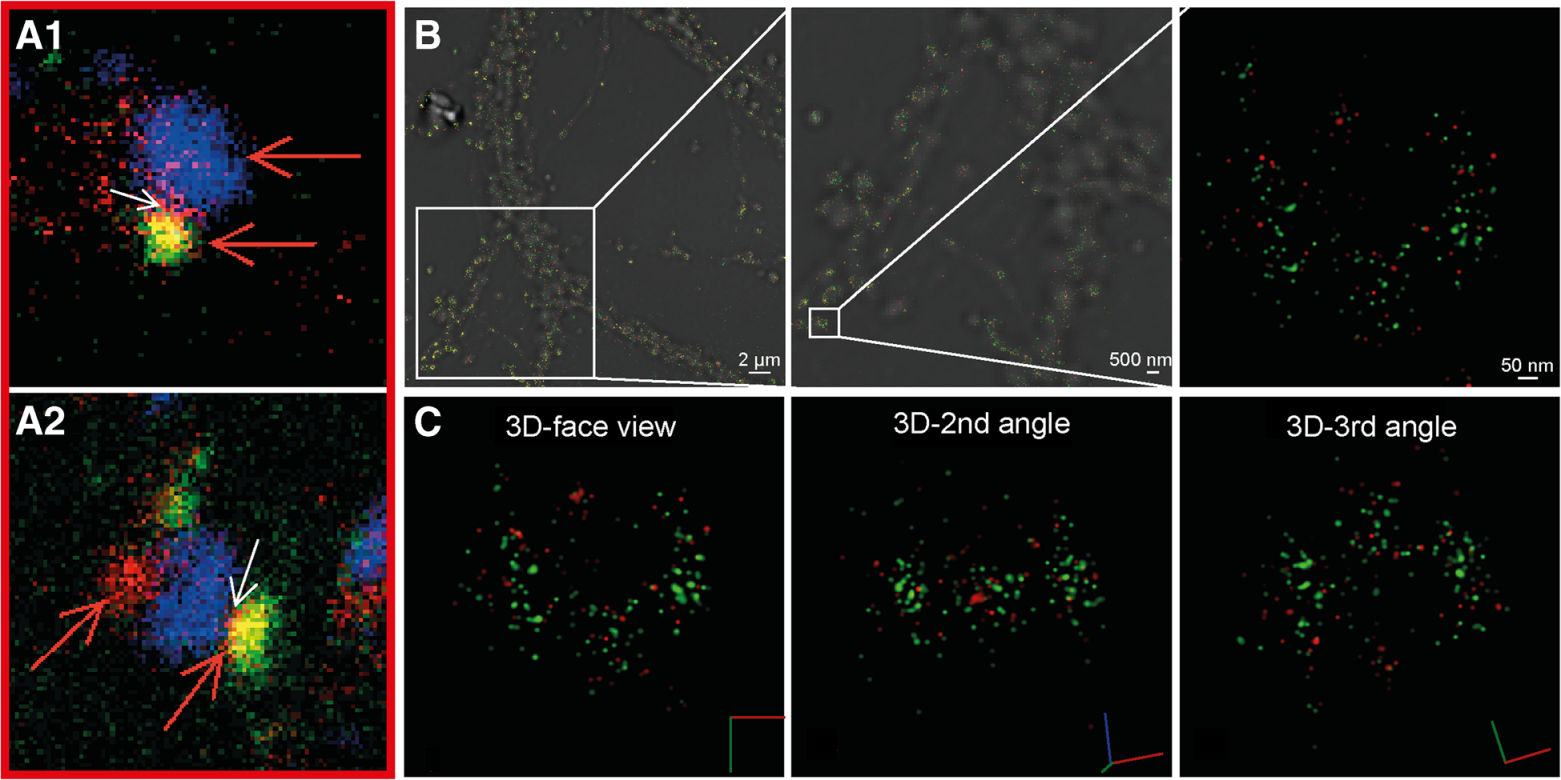
Localization of γ-secretase at the postsynaptic plasma membrane. (**a**) STED images showing localization of γ-secretase (red) very close to the synaptic cleft in the dendritic spines (*white arrows*). Synaptophysin (blue) and PSD95 (green) mark the pre- and postsynaptic sides, respectively. The close-ups are taken from Additional file 1: Figure S5b (a1) and S5d (a2). (**b**) STORM imaging of γ-secretase (*red*) and NMDAR2B (*green*). The image shows a typical STORM image on a brightfield overlay (*left*), with increased magnification in the *middle* and *right panels*, displaying a close-up from one individual postsynaptic membrane region to the right. (**c**) The same postsynaptic membrane region as the right image in (b) is displayed in 3D view from three different angles where *red*, *green* and *blue lines* represent X, Y and Z directions, respectively

To further investigate the distribution of γ-secretase in the pre- and postsynaptic compartments, the samples were quadruple-stained to make a three-color STED image of γ-secretase, synaptobrevin and PSD95, overlayed on a confocal image of the neuronal structure. The latter was visualized with Phalloidin conjugated to Alexa488, as a marker for f-actin. The presence of γ-secretase in both the pre- and postsynaptic sides of the same synapse could thus be confirmed (Fig. 4). A close-up of a typical synapse showed intense γ-secretase staining in the postsynaptic compartment and less intense staining in the presynaptic compartment (Fig. 4b-g). As shown above by STORM and STED analyses, the presence of γ-secretase at different synapses was found to be highly variable (Additional file 1: Figure S6). Some synapses contained only little or no γ-secretase at the presynaptic side (Additional file 1: Figure S5a and c), whereas others contained γ-secretase at both sides of the synapse (Additional file 1: Figure S5b, d and e). Interestingly, the γ-secretase distribution in the postsynaptic compartment in mature synapses was most dense in the side facing the synaptic cleft, i.e. between the postsynaptic density and the synaptic cleft, suggesting that γ-secretase is enriched in the postsynaptic plasma membrane (Additional file 1: Figure S5a-e). STORM analysis, showing close proximity between NMDAR2B and γ-secretase, provided further evidence for the presence of γ-secretase in the postsynaptic membrane (Fig. 5).

### Quantification of γ-secretase staining in the synapse

To investigate whether there were any correlations between γ-secretase and the pre-and postsynaptic markers in the synapse, γ-secretase (GTB) versus synaptophysin or synaptobrevin and PSD95 stainings were quantified in the synapse from both STORM and STED data. γ-Secretase staining showed a positive correlation with both the pre- and postsynaptic markers (Additional file 1: Figure S6).

## Discussion

The enzyme complex γ-secretase is involved in regulated intramembrane proteolytic processing of around 100 substrates including APP, the processing of which leads to formation of the AD associated pathogen Aβ. Most likely, various γ-secretase activities are separated in different cellular compartments, and determining the subcellular location of active γ-secretase is critical for understanding its role in AD pathogenesis. Hitherto, technical limitations have hindered exact determination of its subcellular location and, in addition, most previous studies have been directed to individual γ-secretase components instead of the active enzyme and are thus inconclusive. Here, we took advantage of STORM to image γ-secretase in neurites and synapses of mouse primary hippocampal neurons in 3D with ultra-high resolution, as well as STED to image triple- and quadruple-stained samples. Together, these techniques generated conclusive data showing the presence of γ-secretase in both pre- and postsynaptic compartments, and showed that the enzyme is differently expressed on the two sides of the synapse at different synaptic stages.

The presence of γ-secretase in the presynaptic compartment was obvious both on presynaptic boutons and axon terminals, and the distribution of γ-secretase varied from synapse to synapse. Formation of the presynapse has been suggested to occur in several steps, where pre-boutonal structures, called “primitive axonal synaptic vesicle release sites”, which contain only a few synaptic vesicles, first form in the absence of dendritic contacts. Then, in sequential steps, the axon comes in contact with a dendrite, the bouton grows in size, an active zone is formed and synaptic vesicles dock to the membrane. Eventually a fully developed presynaptic bouton that is in contact with a dendritic spine and contains an active zone, docked synaptic vesicles and a large reserve pool of synaptic vesicles is formed [16]. The finding that both Tau and γ-secretase accumulate at the edges of pre-boutons suggests that γ-secretase is located close to or at the plasma membrane in these structures. In contrast, the majority of γ-secretase in the mature presynaptic boutons was found in intracellular structures, suggesting that the presynaptic location of γ-secretase is dynamic and changes during maturation. The presynaptic marker synaptophysin is located in the membrane of synaptic vesicles and regulates endocytosis of these vesicles [14]. In some synapses, synaptophysin was present in clustered regions, presumably representing docked synaptic vesicles. Interestingly, γ-secretase was absent in these regions. These findings are in line with earlier studies showing that Aβ is not released in a synaptic activity-dependent manner [17] and that synaptic vesicles are not enriched with γ-secretase [18]. Thus it is likely that γ-secretase is located in another type of vesicle within the presynapse, for instance endosomes, autophagosomes and/or exosomes [18, 19].

Dendritic spines of highly variable shapes were observed, which may reflect differences in their function [20]. STORM analysis showed that most of the PSD95-containing spines also contained γ-secretase, and quantitative data showed a higher extent of PSD95 clusters lacking γ-secretase in thin than in thick neurites, which may reflect differences in developmental stages of the synapses. The distribution of γ-secretase in the postsynaptic compartment was more dense than in the presynaptic compartment, although a large synapse to synapse variability was observed. PSD95 was present in one or more clusters per spine head, in agreement with previous studies [15]. The size of the postsynaptic density clusters determined by STORM were variable but typically measured 0.08 × 0.10 μm in two dimensions (Fig. 1b), in agreement with previous studies [15]. Some spines contained γ-secretase throughout the spine head whereas others contained γ-secretase only closely associated to PSD95. Interestingly, in several of the quadruple-stained synapses that stained positive for both PSD95 and synaptophysin, γ-secretase was enriched in the space between the postsynaptic density and the synaptic cleft. Since PSD95 is located only 12–26 nm from the synaptic cleft [12] and anchors to plasma-membrane proteins, these findings suggest that γ-secretase is accumulated in the postsynaptic plasma membrane of mature spines. Similarly, STORM images of certain synapses showed dense γ-secretase staining precisely outside the synaptophysin clustering (active zone), with a distance from the synaptophysin clustering in agreement with the size of the synaptic cleft, giving further support to the presence of γ-secretase in the postsynaptic plasma membrane. In addition, the close proximity between NMDAR2B and γ-secretase observed by STORM analysis supported this notion. The mechanism of oligomeric Aβ synaptotoxicity is not fully elucidated, although proposed mechanisms include binding of oligomeric Aβ to the postsynaptic receptors, including metabotropic glutamate receptor (mGluR5) and the cellular prion protein PrP^c^. It has been proposed that Aβ reduces the lateral mobility of mGluR5, with aberrant effects on receptor functions and loss of dendritic spines [21, 22]. If Aβ is formed directly in the postsynaptic plasma membrane, as our data suggest, the distance to the postsynaptic receptors is indeed close.

The neurons used in this study were grown for 21 days in vitro (DIV) and contain a mixture of synapses at different developmental stages [16]. A large synapse to synapse variability was observed with both STORM and STED, and the proportion of the type of synapses observed with the two imaging methods showed some variation. In all cases, there was a positive correlation between the intensity of γ-secretase labeling and the intensity of the labeling of the synaptic markers (Additional file 1: Figure S6). With STED, there was a relatively higher γ-secretase labeling intensity in the postsynaptic compartment than in the presynaptic compartment. In contrast, with STORM we observed a relatively larger γ-secretase staining intensity in the presynaptic than in the postsynaptic compartments. This discrepancy could be related to the fact that the selection of synapses analyzed differed with the two methods. With STORM, all clusters of synaptophysin or PSD95 at mature dendrites were evaluated, whereas with STED, spines with a clear shaft and spine head that were perpendicular to the light beam were selected. Thus the latter method more stringently finds fully developed, presumably excitatory glutamatergic, spines, whereas STORM analyzed all types of synapses. In summary, our data suggest that γ-secretase is abundant at the presynaptic side during the early stage in the development of presynapses, and the expression of γ-secretase increases in the pre- and postsynaptic compartments as the synapse matures, suggesting a correlation between γ-secretase activity and synapse maturation. Thus it is possible that especially the more mature synapses are responsible for Aβ production.

## Conclusions

We show that γ-secretase is present mostly in intracellular organelles in the presynaptic compartment, whereas it is found in both intracellular organelles and in the plasma membrane of the postsynaptic compartment. The abundance in the pre- and postsynaptic compartments increases with the maturation of the synapse. Our findings indicate that Aβ can be produced at both the pre- and postsynaptic compartments. This study is thus of high importance for further understanding AD pathology, which may be relevant for creating new approaches to develop AD treatments.

## Additional files

Additional file 1: Figure S1. Competition of GTB with active site inhibitor JC18 in hippocampal neurons. **Figure S2.** Negative controls for STORM. **Figure S3.** STORM imaging of the variation of γ-secretase in synapses. **Figure S4.** STED imaging showing the variation of γ-secretase labeling at synapses of triple stained mouse primary hippocampal neurons. **Figure S5.** STED confocal imaging of γ-secretase at the synapses of quadruple-stained mouse primary hippocampal neurons. **Figure S6.** Quantification of γ-secretase staining in synapses from STORM and STED data. (PDF 17769 kb) Additional file 2: Video S1. Labeling of the presynaptic marker synaptophysin (blue) and γ-secretase (red) demonstrates that gamma-secretase is present in the presynapse. (AVI 8637 kb) Additional file 3: Video S3. Clusters of synaptophysin (blue) and γ-secretase (red). (AVI 10380 kb) Additional file 4: Video S2. Labeling of the postsynaptic marker PSD95 (green) and γ-secretase (red) demonstrates that γ-secretase is present in the postsynapse. (AVI 8929 kb) Additional file 5: Video S4. Two proximate clusters of PSD95 (green) and γ-secretase (red). (AVI 11039 kb)
