# Supplementary material for: Super-resolution microscopy reveals γ-secretase at both sides of the neuronal synapse
Source: Acta Neuropathol Commun. 2016 Mar 31;4:29. doi: 10.1186/s40478-016-0296-5 (PMC4818506; doi:10.1186/s40478-016-0296-5)
Supplement: Additional file 1: Figure S1. — Competition of GTB with active site inhibitor JC18 in hippocampal neurons. Figure S2. Negative controls for STORM. Figure S3. STORM imaging of the variation of γ-secretase in synapses. Figure S4. STED imaging showing the variation of γ-secretase labeling at synapses of triple stained mouse primary hippocampal neurons. Figure S5. STED confocal imaging of γ-secretase at the synapses of quadruple-stained mouse primary hippocampal neurons. Figure S6. Quantification of γ-secretase staining in synapses from STORM and STED data. (PDF 17769 kb) [file 40478_2016_296_MOESM1_ESM.pdf]

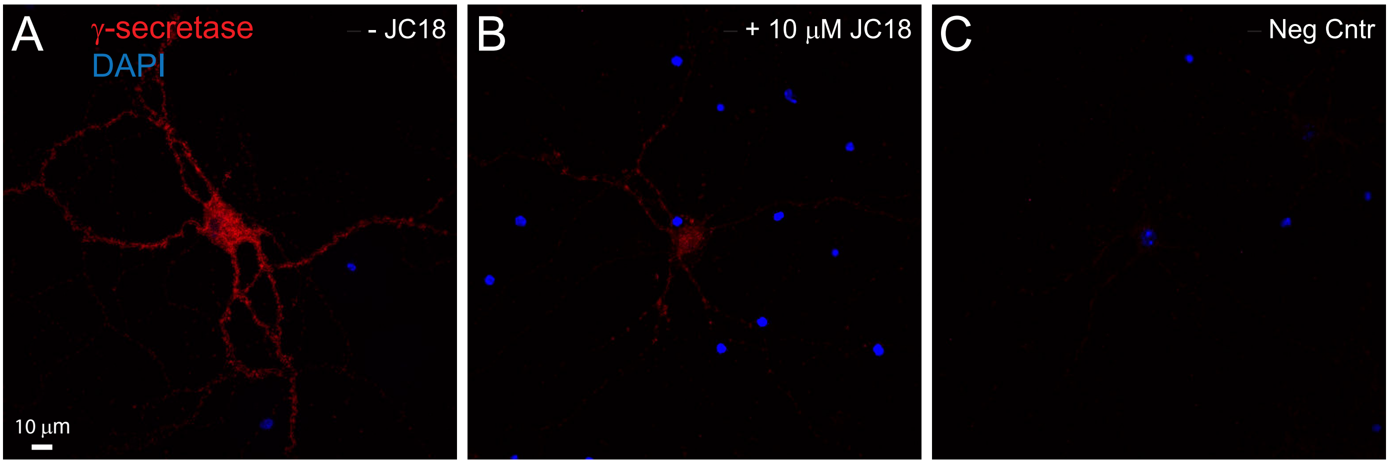

Fig. S1. Competition of GTB with active site inhibitor JC18 in hippocampal neurons. Staining of γ-secretase (red) with the probe GTB and streptavidin in primary hippocampal neurons was performed **(a)** in the absence, or **(b)** in the presence of 10 μM γ-secretase inhibitor JC18. (c) The background staining of streptavidin in the absence of GTB was used as a negative control. The image was acquired in sequential mode on a Nikon A1RSi point scanning confocal inverted microscope using a 60X oil immersion objective with an image size of 1024 X 1024 pixels. Excitation lasers were 405 (DAPI) and 561 (GTB/streptavidin) and a pinhole size corresponding to 1 airy unit (at 561 nm) was used. Unbiased selection of neurons was performed by first choosing images with one representative nuclei from the DAPI channel and then adding the GTB/streptavidin channel. Two to five images were taken at each concentration and representative images are shown.

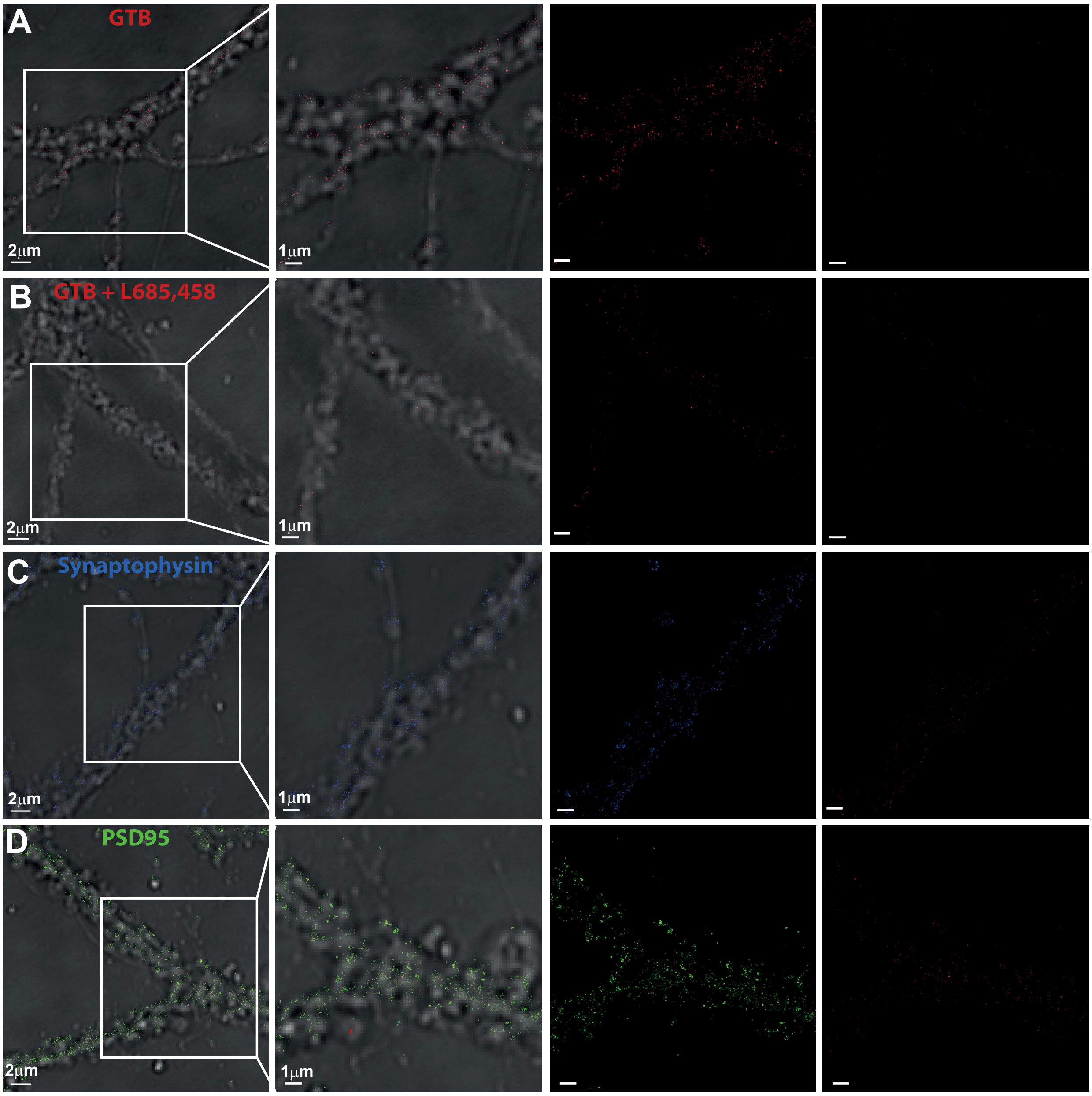

Fig. S2. See legend on the next page.

Fig. S2. Negative controls for STORM. The localization of γ-secretase in the pre-and postsynaptic regions were determined by activator-based STORM, using fluorescent probes containing two types of fluorophores, one activator (Cy3 for the GTB-streptavidin complex and Alexa405 for the secondary antibodies targeting PSD95 or synaptophysin) and one reporter (Alexa647). Two-channel settings were used with 405 and 561 activation lasers while the emission of the reporter obtained by a 647 laser was recorded. Imaging cycles were set to contain one activation frame (i.e. image acquired directly after an activation laser pulse) followed by four reporter frames (i.e. images taken of the reporter emission), to be able to differ between probe-specific and unspecific signals. Since the reporter fluorophores may blink even in the absence of activator, the activator laser light to some extent affects the blinking speed even in the absence of activator fluorophore. Negative controls lacking either the γ-secretase probe (GTB) or the antibody directed to the synaptic marker (synaptophysin or PSD95) or to measure potential cross-talk between the channels were thus also analyzed **(a)**, **(c)** and **(d**). In addition a negative control showing that GTB could be competed by L-685,458 was included **(b)**.

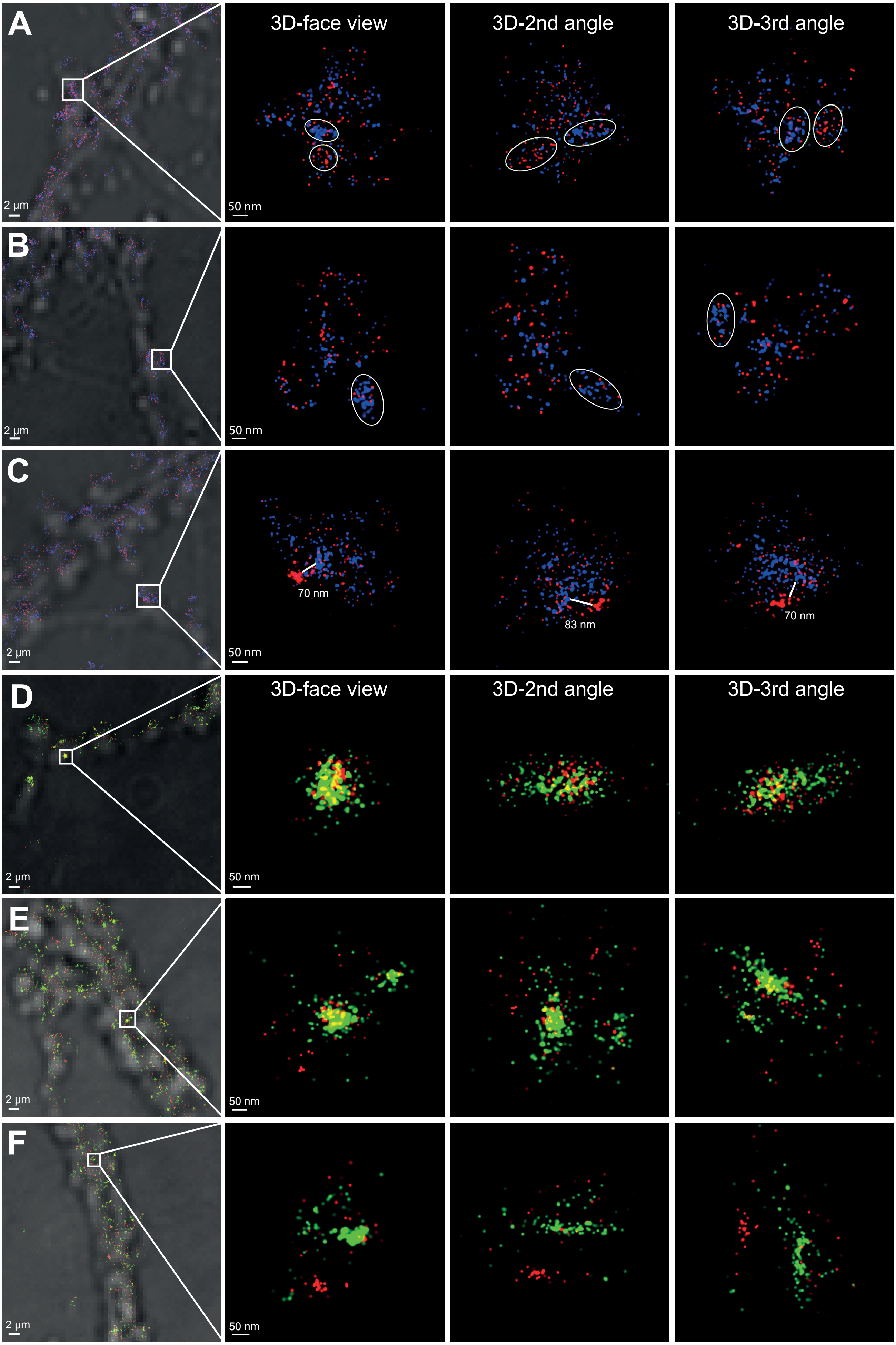

Fig. S3. See legend on the next page.

Fig. S3. STORM imaging of the variation of γ-secretase in synapses. Two-colour, three-dimensional STORM imaging of γ-secretase at the synapse of mouse primary hippocampal neurons. **(a)** Variability of γ-secretase and synaptophysin staining in the presynaptic compartment. Upper and middle panels show that clusters of γ-secretase and synaptophysin were found (marked with white ovals). The lower panel shows clustering of γ-secretase outside the presynaptic compartment. **(b)** Variability of γ-secretase staining in the postsynaptic compartment. The upper panel shows an example of a postsynapse with similar distribution of γ-secretase and PSD 95, the middle panel shows two PSD95 clusters in close proximity. The lower panel shows one PSD95 cluster and one γ-secretase cluster that are separated in space.

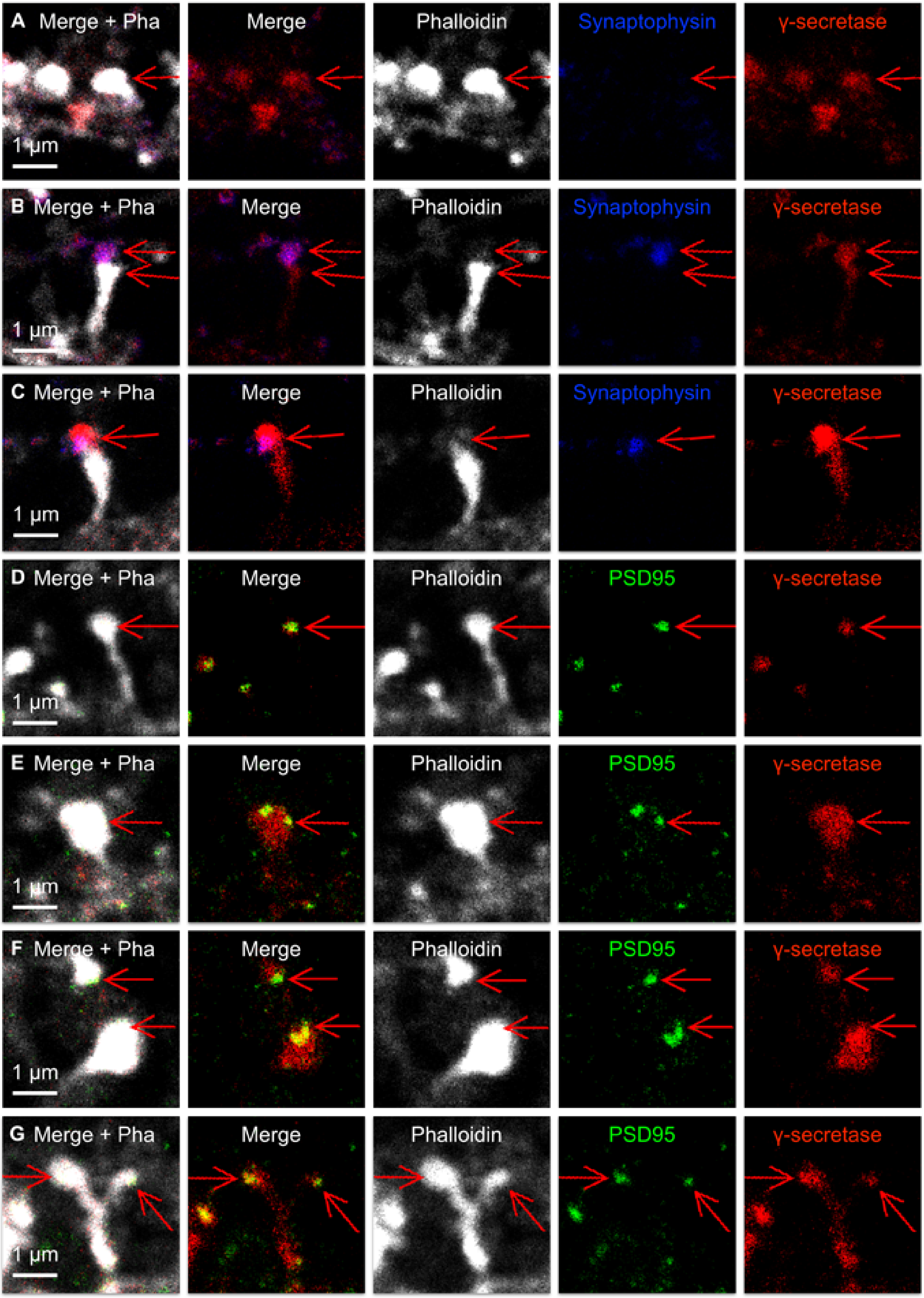

Fig. S4. See legend on next page.

Fig. S4. STED imaging showing the variation of γ-secretase labeling at synapses of triple stained mouse primary hippocampal neurons. The neurite structure is visualized by phalloidin as a marker for f-actin (white). **(a)** Localization of γ-secretase (red) in dendritic spines without any presynaptic synaptophysin (blue) staining. **(b)** Localization of γ-secretase (red) both in a dendritic spine (white) and in the presynapse, visualized by synaptophysin (blue). **(c)** Localization of dense labeling of γ-secretase (red) at the presynapse, visualized by synaptophysin (blue). **(d)** Localization of dense labeling of γ-secretase (red) at dendritic spines, visualized by phalloidin (white) and PSD95 (green). **(e)** Image showing γ-secretase (red) in the whole head of a postsynaptic dendritic spine. Large dendritic spines are sometimes displaying multiple dense areas of PSD95 (green). **(f)** Image showing γ-secretase (red) in two adjacent dendritic spines that might face the same axonal bouton, with small amount of γ-secretase in the area between the two dendritic spines, presumably reflecting the presynapse. **(g)** Image showing γ-secretase (red) in a double-headed dendritic spine.

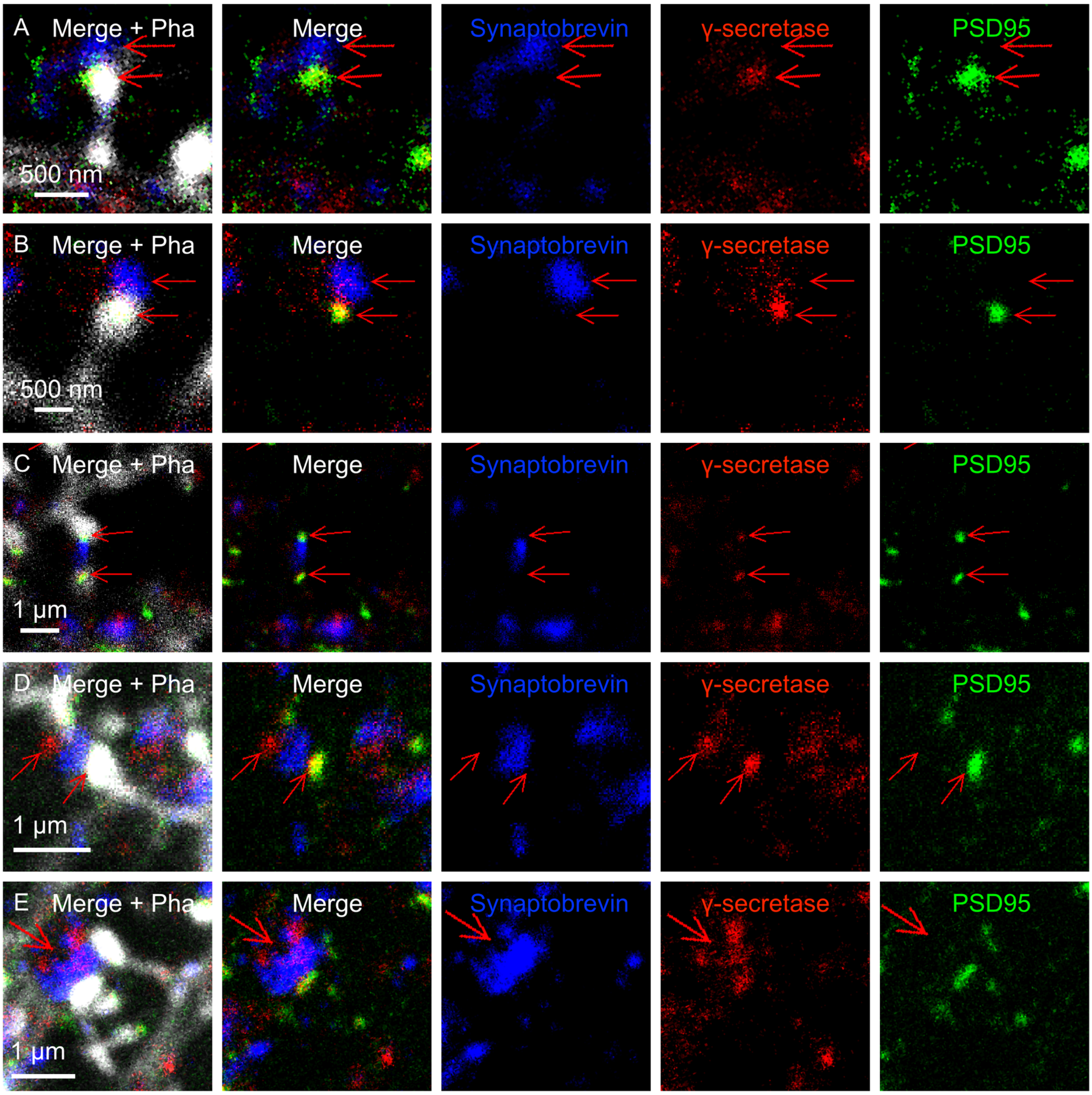

Fig S5. See legend on next page.

Fig S5. STED confocal imaging of γ-secretase at the synapses of quadruple-stained mouse primary hippocampal neurons. γ-Secretase, postsynaptic PSD95 and presynaptic synaptobrevin were imaged by STED microscopy and dendritic spine structure of Phalloidin, as a marker for f-actin, was overlaid in a non-STED confocal channel (white). (**a**) Image showing a typical dendritic spine where γ-secretase is found at the postsynapse, inside the dendritic spine head (lower arrow), co-localizing with PSD95 (green). **(b)** Image showing a dendritic spine where γ-secretase is mainly found at the postsynapse in the dendritic spine head (lower arrow), co-localized with PSD95 (green), and some γ-secretase in the presynapse (upper arrow), co-localized with synaptobrevin (blue). **(c)** Image showing γ-secretase (red) in two adjacent dendritic spines, which face the same axonal bouton (blue). **(d)** Image showing γ-secretase (red) in two adjacent dendritic spines that face the same axonal bouton (blue). Accumulation of γ-secretase (red) is found at or in close connection to the presynapse (left arrow). **(e)** Large amount of γ-secretase at presynapses at dense areas of synaptobrevin (blue).

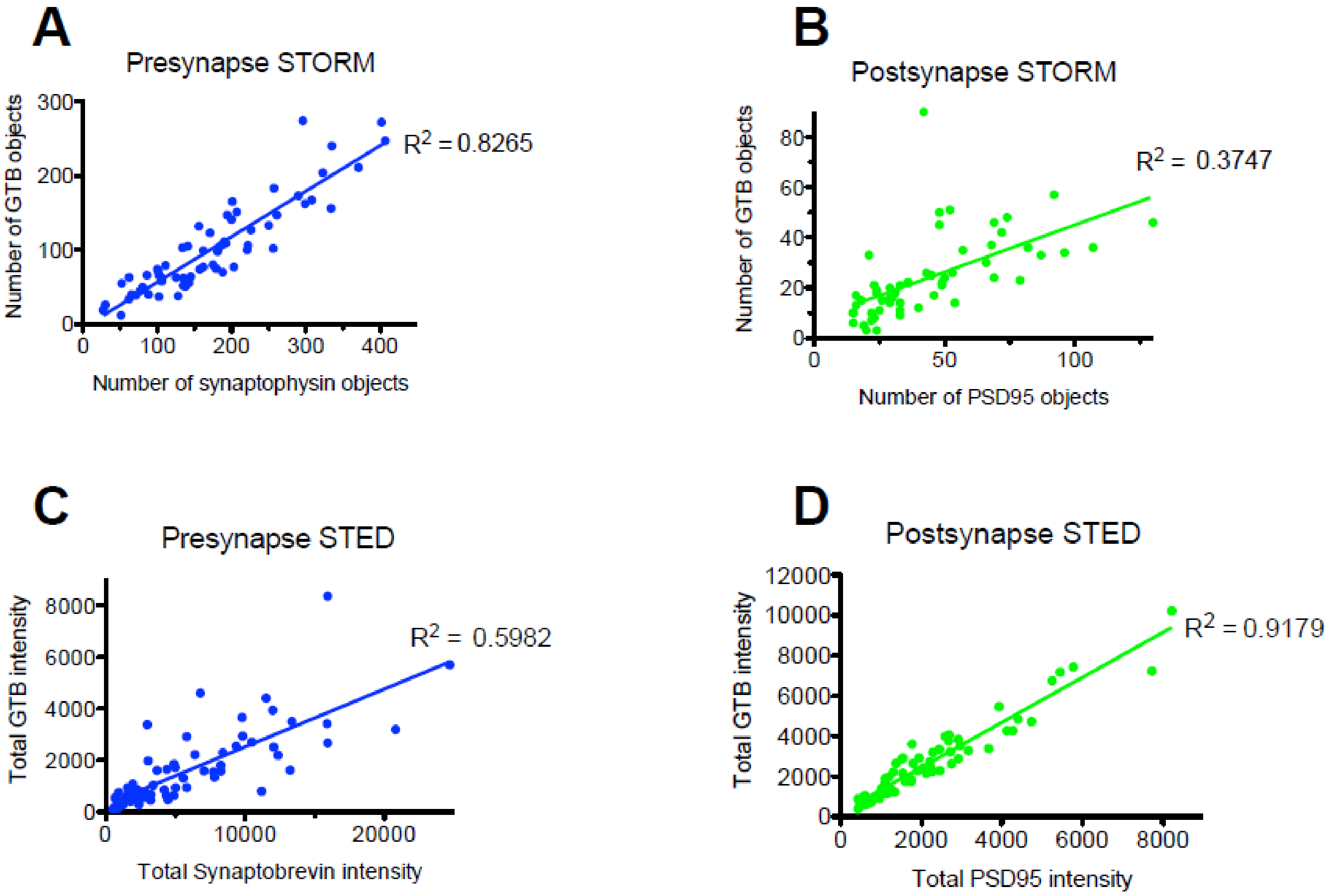

Fig S6. See legend on next page.

Fig S6. Quantification of γ-secretase staining in the synapse. The staining of γ-secretase (GTB) versus the presynaptic marker (synaptophysin or sybaptobrevin) or postsynaptic marker (PSD95) were quantified from both STORM and STED data. The number of signals in STORM images were quantified by General Analysis in NIS elements (Nikon). GTB signals were calculated from 60 PSD95 clusters in one STORM image and from 66 synaptophysin clusters in another STORM image. The number of GTB signals was plotted versus the number of synaptophysin **(a)** or PSD95 signals **(b).** The background of the GTB signals, calculated as the mean values ± SD from 10 synaptophysin or PSD95 clusters in samples that lacked GTB were 15±6 and 7.1 ±3.2 for synaptophysin and PSD95 stainings, respectively. Quadruple stained STED micrographs were calculated for total fluorescence intensity in 70 pre-postsynapse pairs by LAS AF Version 2.6.3 build 8173 Software (Leica Microsystems CMS GmbH). Background fluorescence was subtracted. Fluorescence intensity of GTB versus synaptobrevin **(c)** and PSD95 **(d)** was plotted. Plotting and statistical analysis was performed by GraphPad Prism 5.0d (GraphPad Software, Inc.).
